# Immunohistochemical analysis revealed the expression of bone morphogenetic proteins-4, 6, 7, and 9 in human induced membrane samples treated with the Masquelet technique

**DOI:** 10.1186/s13018-022-02922-y

**Published:** 2022-01-15

**Authors:** Takahiro Niikura, Takahiro Oda, Naoe Jimbo, Masato Komatsu, Keisuke Oe, Tomoaki Fukui, Tomoyuki Matsumoto, Shinya Hayashi, Takehiko Matsushita, Tomoo Itoh, Ryosuke Kuroda

**Affiliations:** 1 Department of Orthopaedic Surgery, Kobe University Graduate School of Medicine, 7-5-1 Kusunoki-cho, Chuo-ku, Kobe, 650-0017 Japan; 2 Department of Diagnostic Pathology, Kobe University Graduate School of Medicine, Kobe, Japan

**Keywords:** Bone defect, Bone morphogenetic protein, Immunohistochemistry, Induced membrane, Masquelet technique

## Abstract

**Background:**

Induced membrane (IM) is the key component of Masquelet reconstruction surgery for the treatment of bone defects. IM is formed around the cement spacer and is known to secrete growth factors and osteoinductive factors. However, there is limited evidence available concerning the presence of osteoinductive factors in IM. This study aimed to investigate the existence of bone morphogenetic proteins (BMPs) in IM harvested from patients during the treatment of bone defects using the Masquelet technique.

**Methods:**

This study involved six patients whose bone defects had been treated using the Masquelet technique. The affected sites were the femur (*n* = 3) and the tibia (*n* = 3). During the second-stage surgery, 1 cm^2^ pieces of IM were harvested. Histological sections of IM were immunostained with anti-BMP-4, 6, 7, and 9 antibodies. Human bone tissue served as the positive control.

**Results:**

The presence of BMP-4, 6, 7, and 9 was observed in all IM samples. Further, immunolocalization of BMP-4, 6, 7, and 9 was observed in blood vessels and fibroblasts in all IM samples. Immunolocalization of BMP-4, 6, 7, and 9 was also observed in bone tissue within the IM in one sample, in which osteogenesis inside the IM was observed.

**Conclusions:**

This study showed that osteoinductive factors BMP-4, 6, 7, and 9 were present in the IM harvested from patients, providing evidence indicating that the Masquelet technique effectively contributes to healing large bone defects. Therefore, it may be possible for surgeons to omit the addition of BMPs to bone grafts, given the endogenous secretion of BMPs from the IM.

## Background

In orthopedic surgery, the reconstruction of a large bone defect due to trauma, infection, or tumors remains challenging. Distraction osteogenesis using the Ilizarov method [1, 2] and vascularized fibula grafts [3, 4] continue to be limited treatment options. The Masquelet technique, also known as the induced membrane (IM) technique, provides a third option for reconstruction of large bone defects [5–7], and is comprised of a two-staged surgery. In the first stage, bone defects due to debridement are filled with a polymethyl methacrylate (PMMA) cement spacer. A bioactive IM then forms around the cement spacer. After the IM has formed, the cement is removed, and autologous bone grafting is performed in the space of the bone defect surrounded by the IM. The latter serves as a conduit for cells and provides a favorable environment for bone graft osseointegration. IM formed around the cement spacer is a key component of Masquelet reconstruction surgery [5–8]. Previous studies have shown that IM possesses osteogenic and osteoinductive properties, and is richly vascular [9–16].

Bone morphogenetic proteins (BMPs) are representative osteoinductive factors. They stimulate the proliferation and differentiation of mesenchymal stem cells or chondro-/osteo-progenitor cells involved in endochondral or intramembranous ossification [17, 18]. Furthermore, the expression of BMPs during fracture repair has also been reported [19–21]. Endogenous BMPs are important for bone regeneration and repair [22]. Exogenous BMPs such as BMP-2 and 7 have been applied to promote bone regeneration and repair for open fractures and non-union [23, 24]. Recently, BMPs have been utilized as osteoinductive factors as per the ‘diamond concept’ when using the Masquelet technique [25–28].

The expression of BMP-2 protein has been detected using immunohistochemistry, enzyme-linked immunosorbent assay, and western blotting in human [15, 29] and animal [9–11, 30] IM samples. The gene expression of BMP-2, 3b, 6, 7, 10, and 14 has also been detected in human IM samples [29, 31]. However, the presence of BMP proteins other than BMP-2 has not yet been observed using immunohistochemistry in IM samples. Therefore, this study aimed to investigate the presence of BMPs (BMP-4, 6, 7, and 9) using histological samples of human IM.

## Methods

### Ethical approval

This study was performed in accordance with the ethical standards laid down by the 1964 Helsinki Declaration and its later amendments, and was approved by the Ethics Committee of our university. Due to the study’s retrospective design, the requirement for informed consent was waived.

### Patient inclusion

Six patients from our department whose bone defects had been treated using the Masquelet technique were included. The affected sites were the femur (*n* = 3) and the tibia (*n* = 3).

### Histological specimens

During the second surgery (removal of the cement and bone grafting), 1 cm^2^ pieces of the IM that were in contact with the cement spacer were harvested and immersed in 10% neutral buffered formalin. Samples were embedded in paraffin, and histological sections (4 µm in thickness) were made.

### Histological analyses

Histological sections were stained with hematoxylin and eosin and analyzed by two clinical pathologists. The number of blood vessels per 1 mm^2^ within the IM was counted in locations with the highest capillary density. Histological findings of inflammation, foreign body reaction, and fibrosis were assessed using a semi-quantified grading scale ranging from 0 to 3, with grade 3 indicating the highest degree of inflammation, foreign body reaction, and fibrosis, and grade 0 indicating an absence of such findings.

### Immunohistochemistry

After deparaffinization, the sections were incubated overnight at 4 °C with anti-BMP4 primary antibody (1:100 dilution, GTX100875, GeneTex Inc., Hsinchu City, Taiwan), anti-BMP6 primary antibody (1:100 dilution, ab155963, Abcam, Cambridge, MA, USA), anti-BMP7 primary antibody (1:100 dilution, ab84684, Abcam), or anti-BMP9 primary antibody (1:100 dilution, ab35088, Abcam), and subsequently treated with peroxidase-labeled anti-rabbit immunoglobulin (Histofine® Simple Stain MAX PO, Nichirei Bioscience, Tokyo, Japan) at room temperature for 60 min. The signal was observed as the development of a brown reaction product with the peroxidase substrate 3,3′-diaminobenzidine (Histofine® Simple Stain 3,3-Diaminobenzidine Solution, Nichirei Bioscience). The sections were counterstained with hematoxylin and examined using a BZ-X700 confocal microscope (Keyence Corporation, Osaka, Japan). Phosphate-buffered saline (PBS) was used instead of primary antibodies to stain the negative control samples. Formalin-fixed paraffin-embedded human tissue sections (catalog number CS812148, case ID CU0000012835, 63-year-old, male, bone, distal femur) were obtained from OriGene Technologies (Rockville, MD, USA), and were used as the positive control.

### Clinical data

Data concerning patient sex, age, morbidity accounting for the bone defect, free flap application to the affected limb, affected site (bone), impregnation of antibiotics to the cement spacer, duration of cement placement, history of smoking, and a history of diabetes mellitus (DM) or peripheral artery disease (PAD) were obtained from medical records. The duration of cement placement was defined as the number of days from the first-stage surgery in which the cement spacer was placed to the second-stage surgery in which the cement spacer was removed and bone grafting was performed. The radiographic apparent bone gap (RABG) [32] was measured to determine the size of each patients’ bone defect using plain radiographs. Bony union in the enrolled patients was assessed radiologically and clinically. The time point of bony union assessment was set at 6 months after the second-stage surgery (autologous bone grafting). Radiological bony union was defined as corticalization of the grafted bone and absence of a gap between the grafted and original bone, observed in three or four cortices using orthogonal (anteroposterior and mediolateral) plain radiographs. Clinically, bony union was defined as the absence of pain on full weight-bearing. Bony union was defined as the achievement of both radiological and clinical bony union. Three experienced orthopedic trauma surgeons individually assessed bony union, which was considered to have been achieved when at least two surgeons agreed that it had occurred.

## Results

### Patient characteristics

Five men and one woman were included in the study (Table 1). The mean age was 53.0 ± 11.1 years (range, 42–72). The morbidities accounting for the bone defects were osteomyelitis (3 patients), infected nonunion (1 patient), non-infected nonunion (1 patient), and severely comminuted open fracture (1 patient). The femur (*n* = 3 patients) and the tibia (*n* = 3 patients) were the affected sites. The RABG was 8.5 ± 3.1 cm (range, 2.7–10.4). The mean cement placement duration was 77.5 ± 31.3 days (range, 46–126). Three patients underwent free flap surgery. Five patients received antibiotic impregnation into the cement. Three patients had a history of smoking, and no patients had a history of DM or PAD. All patients achieved a bony union.

**Table 1:** Patient characteristics

Case	Sex	Age	Affected site	Morbidity accounting for the bone defect	RABG (cm)	Duration of the cement placement (days)	Free flap surgery	Antibiotics within the cement	Smoking	DM	PAD
1	M	58	Femur	Uninfected nonunion	2.7	46	−	−	+	−	−
2	M	51	Femur	Osteomyelitis	10.4	54	−	+	+	−	−
3	F	72	Femur	Osteomyelitis	10.2	56	−	+	−	−	−
4	M	43	Tibia	Severely comminuted open fracture	10.1	83	+	+	+	−	−
5	M	52	Tibia	Osteomyelitis	7.2	126	+	+	−	−	−
6	M	42	Tibia	Infected nonunion	10.3	100	+	+	−	−	−

*DM* diabetes mellitus, *F* female, *M* male, *PAD* peripheral artery disease, *RABG* radiographic apparent bone gap

### Histological findings

IM formation was confirmed histologically in all patients; the histological findings are summarized in Table 2. Blood vessel formation was noted in all patients. The mean number of blood vessels per 1 mm^2^ was 35.0 ± 20.4 (range, 15–70). Inflammation, foreign body reaction, and fibrosis were observed in all patients. The histological grading of inflammation, fibrosis, and foreign body reaction is shown in Table 2. Osteogenesis inside the IM was observed in one patient. A two-layered structure was noted in all patients. A synovial-like structure at the surface that was in contact with the cement was identified in three patients, while fibrin deposition was observed in four patients.

**Table 2:** Summary of the histological findings

Case	Sex	Age	Affected site	Blood vessel counts per 1 mm^2^	Inflammation	Foreign body reaction	Fibrosis	Osteogenesis within the membrane	Two-layer structure	Synovial-like structure at the surface	Fibrin deposition
1	M	58	Femur	70	1	2	1	−	+	+	−
2	M	51	Femur	20	1	3	2	−	+	−	+
3	F	72	Femur	30	2	1	1	−	+	+	+
4	M	43	Tibia	55	1	1	2	+	+	+	−
5	M	52	Tibia	15	2	2	1	−	+	−	+
6	M	42	Tibia	20	2	3	1	−	+	−	+

The degree of inflammation, foreign body reaction, and fibrosis was graded from 0 to 3

*M* male, *F* female

### Immunohistochemical findings

**Fig. 1:**
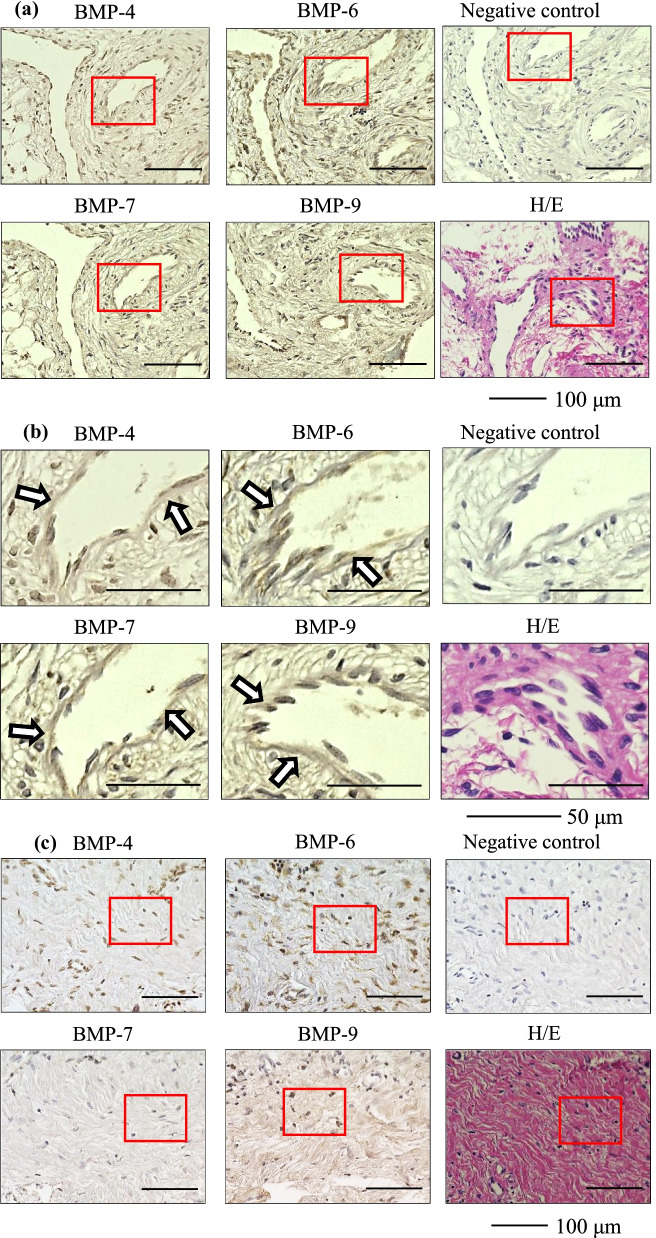

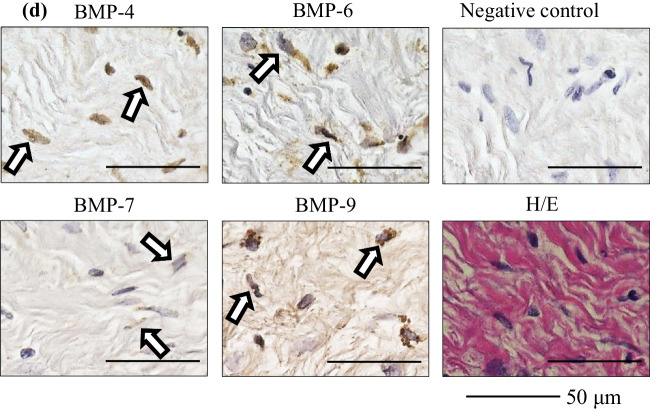
Representative immunohistochemical images of the induced membrane (Case 3, femur) **a** blood vessel, low-power field, **b** blood vessel, high-power field, **c** fibroblast, low-power field, **d** fibroblast, high-power field. The arrows indicate immuno-positive staining. *BMP* bone morphogenetic protein, *H/E* hematoxylin and eosin

**Fig. 2:**
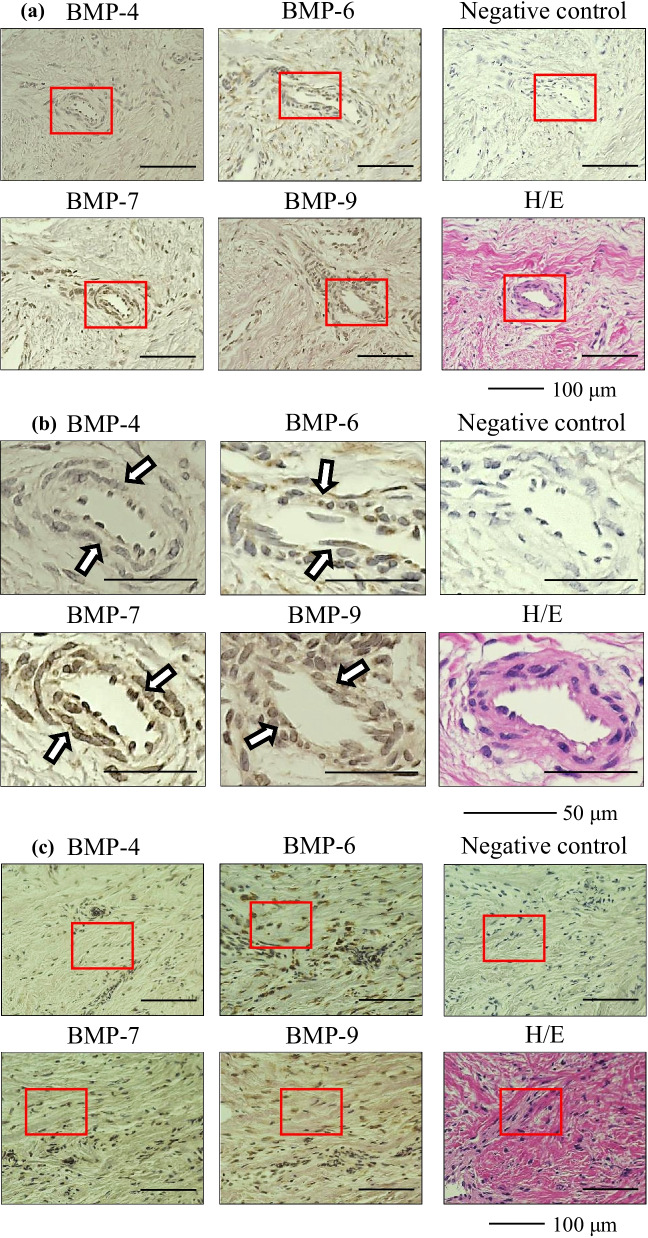

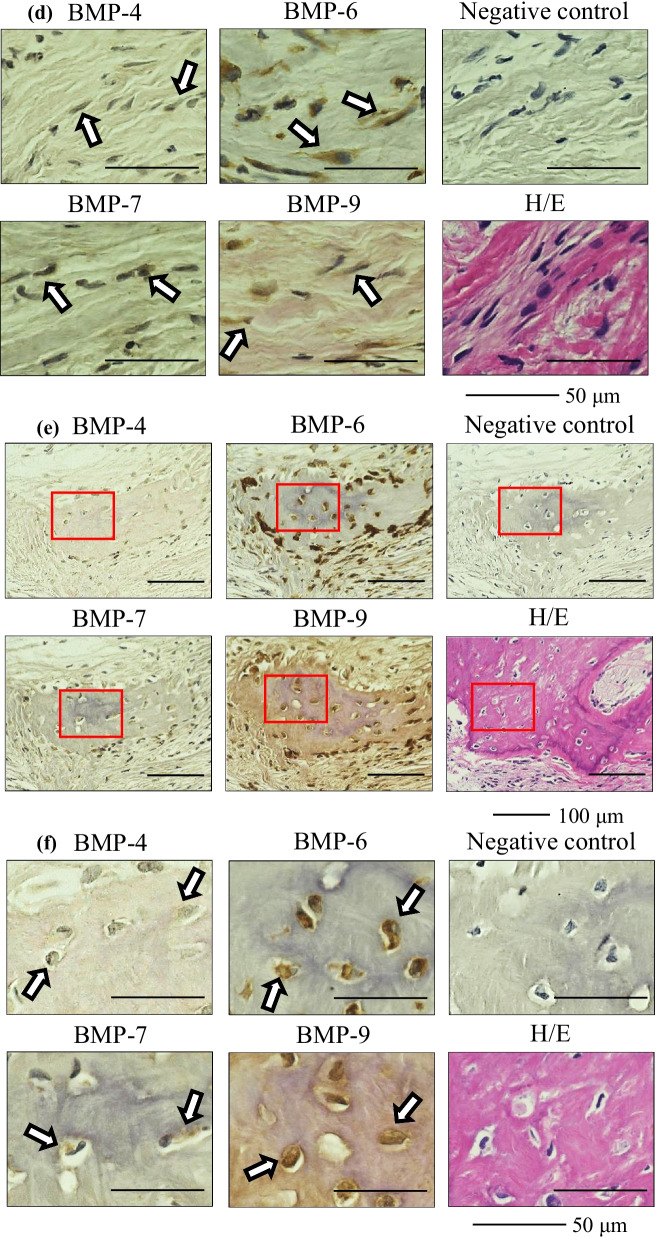
Representative immunohistochemical images of the induced membrane (Case 4, tibia) **a** blood vessel, low-power field, **b** blood vessel, high-power field, **c** fibroblast, low-power field, **d** fibroblast, high-power field, **e** bone inside the induced membrane, low-power field, **f** bone inside the induced membrane, high-power field. The arrows indicate immuno-positive staining. *BMP* bone morphogenetic protein, *H/E* hematoxylin and eosin

**Fig. 3:**
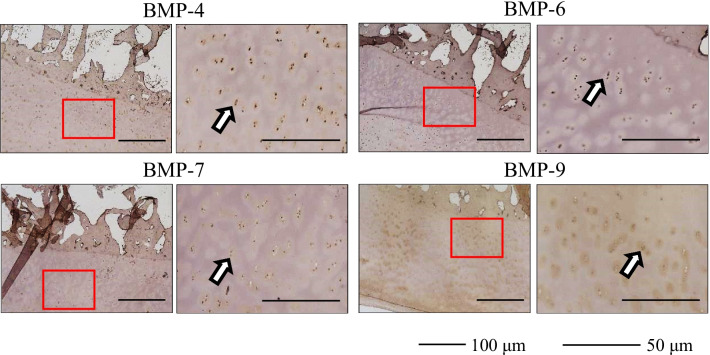
Immunostaining of human bone tissue as a positive control. Left side, low-power field; right side, high-power field. The arrows indicate immuno-positive staining. *BMP* bone morphogenetic protein

The immunohistochemical findings are summarized in Table 3. The presence of BMP-4, 6, 7, and 9 was observed in all IM samples. Representative immunohistochemical images are shown in Fig. 1 (Case 3, femur) and Fig. 2 (Case 4, tibia). Immunolocalization of BMP-4, 6, 7, and 9 was observed in blood vessels (Figs. 1a, b, 2a, b) and in fibroblasts (Figs. 1c, d, 2c, d). These findings were observed in all IM samples. Immunolocalization of BMP-4, 6, 7, and 9 was also observed in the bone within the IM in one sample in which osteogenesis inside the IM was observed (Fig. 2e, f). Finally, immunostaining of human bone tissue as a positive control demonstrated positive immunoreactivity for BMP-4, 6, 7, and 9 (Fig. 3).

**Table 3:** Summary of the immunohistochemical findings

	Blood vessel	Fibroblast	Bone
BMP-4	6/6	6/6	1/6
BMP-6	6/6	6/6	1/6
BMP-7	6/6	6/6	1/6
BMP-9	6/6	6/6	1/6

6/6 means that immunolocalization of the BMPs was observed in the blood vessels and fibroblasts of all samples, out of a total 6 samples

1/6 means that immunolocalization of the BMPs was observed in the bone within the induced membrane in one sample, in which osteogenesis inside the induced membrane was observed

*BMP* bone morphogenetic protein

## Discussion

Recent advances in the Masquelet technique have included innovations using different materials instead of PMMA cement to induce the IM, different techniques of autograft harvesting and placement, and using bone substitutes to supplement the autograft [33]. Histological analyses have also been performed and factors affecting to the vascularity of the IM have been reported [34]. Vitiello et al. [35] recently reported foreign body reaction and IM formation following silver-coated knee megaprosthesis reconstruction.

This study confirmed the presence of BMP-4, 6, 7, and 9 in IMs using human samples, and showed that these BMPs can serve as osteoinductive factors in the treatment of patients with bone defects using the Masquelet technique. This finding helps to clarify the mechanism through which IM promotes bone regeneration and repair in the treatment of bone defects using the Masquelet technique. Therefore, the presence of these BMPs as osteoinductive factors in IM may lead surgeons to omit the addition of exogenous BMPs to bone grafts. However, one animal study showed that the IM technique with BMP and a synthetic scaffold could heal a rat femoral critical size defect [36]. This finding suggested that the addition of exogenous BMP to the IM technique was also an effective option.

BMPs constitute the largest subdivision of the transforming growth factor-β (TGF-β) family of ligands, with nearly 30 distinct human proteins bearing the name [17, 18]. Among the various BMPs, we selected four for this study, based on the following reasons.

BMP-4 belongs to the same subgroup of bone-inducing BMPs as BMP-2, based on the homology of their amino acid sequences. BMP-2 is known to be the most representative osteoinductive factor and has been reported to be widely used clinically to treat bone fracture or nonunion [23, 37, 38]. BMP-4 has been detected along with BMP-2 in the area of endochondral ossification, particularly in the matrix between the newly formed osteoid in human fracture callus [19]. The expression of BMP-4 and Noggin, a major BMP antagonist in tissues, is highlighted in the newly formed callus tissue, thereby confirming the central role of BMP signaling in bone fracture repair [20].

BMP-7 has been clinically applied to treat nonunion of fractures. In a randomized controlled trial, the efficacy of recombinant human BMP-7 (rhBMP-7) in tibial nonunion involving 124 patients who received either autologous bone grafting or a device containing rhBMP-7 was tested [24]. The bone healing rate was found to be inferior in the rhBMP-7 treated group, albeit not statistically significant, and the bone healing capacity of rhBMP-7 was assessed as comparable to that of autologous bone grafting. The United States Food and Drug Administration and the European Medicines Agency have approved rhBMP-7 as a “humanitarian use device” for tibial nonunion. In addition, rhBMP-7 has been used off-label for various indications, including nonunion of the scaphoid, humerus, and clavicle [39–42].

BMP-6 is a paralog of BMP-7 with 87% similarity in their amino acid sequences. BMP-6 is more potent in promoting osteoblast differentiation in vitro and in inducing bone regeneration in vivo when compared with its closely related BMP-7 paralog. This is because of the reversible binding of BMP-6 to Noggin where, unlike BMP-7, BMP-6 may dissociate from Noggin and escape Noggin inhibition [43]. A novel rhBMP-6 containing osteogenic device aimed to accelerate bone regeneration has been developed and is being tested in clinical trials [18, 44, 45].

BMP-9 is a recent discovery in the BMP family. BMP-9 is resistant to Noggin, thus facilitating a more robust cellular differentiation of osteoprogenitor cells into preosteoblasts and osteoblasts [46]. It has been reported that BMP-9 stimulates callus formation in osteoporotic rats during fracture healing [47]. Aside from its osteogenic activity, BMP-9 is involved in a broad range of biological functions, including stem cell differentiation, angiogenesis, neurogenesis, tumorigenesis, and metabolic functions [48]. BMP-9 is likely to be a promising alternative to clinically available BMPs.

We found BMP-4, 6, 7, and 9 were present in all the human IM samples. In addition to immunolocalization of BMP-4, 6, 7, and 9 in blood vessels and fibroblasts, they were also observed within the bone inside the IM in one sample, in which osteogenesis inside the IM was observed. However, this histological finding of osteogenesis inside the IM was found in only one of the six samples. Other studies have also reported this finding in limited samples [14, 29]. We consider that BMP-4, 6, 7, and 9 present inside the IM and contributing to osteoinduction is an important finding.

The Masquelet technique is frequently applied to the lower extremities rather than to the upper extremities. The tibia and femur are long bones of the lower extremities and frequently become subject to bone defects. The volume of the surrounding soft tissue is greater in the femur than in the tibia, and can be considered to reflect vascularity, thereby potentially affecting the formation and properties of the IM. Therefore, we included patients with bone defects in the tibia and femur in this study. Our study results showed that IMs harvested from patients with tibia and femur bone defects expressed BMP-4, 6, 7, and 9.

The strength of this study was the use of human IM samples harvested from patients with bone defects treated using the Masquelet technique. In addition, we reported the presence of BMPs not previously found at the protein or the mRNA level. A limitation of this study was that specimens were harvested from a limited number of patients. Moreover, this was a retrospective study conducted at a single institution, and could therefore be susceptible to selection bias and limited generalizability.

Future studies are needed to further confirm findings. The BMP family, which plays a central role in osteoinduction, comprises many growth factors, while other factors also affect osteoinduction. Therefore, we intend to investigate these other BMPs and osteoinductive factors to further clarify the osteoinductive effect of the IM using human IM samples.

## Conclusion

Osteoinductive factors, BMP-4, 6, 7, and 9 were found to be present in the IM of patients treated using the Masquelet technique.

## Data Availability

Data are available upon reasonable request by contacting the corresponding author.

## Abbreviations

BMP: Bone morphogenetic protein
DM: Diabetes mellitus
IM: Induced membrane
PMMA: Polymethyl methacrylate
PAD: Peripheral artery disease
RABG: Radiographic apparent bone gap

## References

[1] Aktuglu K, Erol K, Vahabi A (2019). Ilizarov bone transport and treatment of critical-sized tibial bone defects: a narrative review. J Orthop Traumatol.

[2] Demiralp B, Ege T, Kose O, Yurttas Y, Basbozkurt M (2014). Reconstruction of intercalary bone defects following bone tumor resection with segmental bone transport using an Ilizarov circular external fixator. J Orthop Sci.

[3] Cano-Luís P, Andrés-Cano P, Ricón-Recarey FJ, Giráldez-Sánchez MA (2018). Treatment of posttraumatic bone defects of the forearm with vascularized fibular grafts. Follow up after fourteen years. Injury.

[4] Tanaka K, Maehara H, Kanaya F (2012). Vascularized fibular graft for bone defects after wide resection of musculoskeletal tumors. J Orthop Sci.

[5] Masquelet AC, Begue T (2010). The concept of induced membrane for reconstruction of long bone defects. Orthop Clin N Am.

[6] Karger C, Kishi T, Schneider L, Fitoussi F, Masquelet AC, French Society of Orthopaedic Surgery and Traumatology (SoFCOT) (2012). Treatment of posttraumatic bone defects by the induced membrane technique. Orthop Traumatol Surg Res.

[7] Masquelet AC (2017). Induced membrane technique: pearls and pitfalls. J Orthop Trauma.

[8] Giannoudis PV, Harwood PJ, Tosounidis T, Kanakaris NK (2016). Restoration of long bone defects treated with the induced membrane technique: protocol and outcomes. Injury.

[9] Pelissier P, Masquelet AC, Bareille R, Pelissier SM, Amedee J (2004). Induced membranes secrete growth factors including vascular and osteoinductive factors and could stimulate bone regeneration. J Orthop Res.

[10] Christou C, Oliver RA, Yu Y, Walsh WR (2014). The Masquelet technique for membrane induction and the healing of ovine critical sized segmental defects. PLoS ONE.

[11] Wang X, Wei F, Luo F, Huang K, Xie Z (2015). Induction of granulation tissue for the secretion of growth factors and the promotion of bone defect repair. J Orthop Surg Res.

[12] Gouron R, Petit L, Boudot C, Six I, Brazier M, Kamel S (2017). Osteoclasts and their precursors are present in the induced-membrane during bone reconstruction using the Masquelet technique. J Tissue Eng Regen Med.

[13] Yılmaz O, Özmeriç A, Alemdaroğlu KB, Celepli P, Hücümenoğlu S, Şahin Ö (2018). Effects of concentrated growth factors (CGF) on the quality of the induced membrane in Masquelet’s technique—an experimental study in rabbits. Injury.

[14] Aho OM, Lehenkari P, Ristiniemi J, Lehtonen S, Risteli J, Leskelä HV (2013). The mechanism of action of induced membranes in bone repair. J Bone Joint Surg Am.

[15] Cuthbert RJ, Churchman SM, Tan HB, McGonagle D, Jones E, Giannoudis PV (2013). Induced periosteum a complex cellular scaffold for the treatment of large bone defects. Bone.

[16] Gindraux F, Rondot T, de Billy B, Zwetyenga N, Fricain JC, Pagnon A (2017). Similarities between induced membrane and amniotic membrane: novelty for bone repair. Placenta.

[17] Lowery JW, Rosen V (2018). The BMP pathway and its inhibitors in the skeleton. Physiol Rev.

[18] Dumic-Cule I, Peric M, Kucko L, Grgurevic L, Pecina M, Vukicevic S (2018). Bone morphogenetic proteins in fracture repair. Int Orthop.

[19] Kloen P, Di Paola M, Borens O, Richmond J, Perino G, Helfet DL (2003). BMP signaling components are expressed in human fracture callus. Bone.

[20] Yoshimura Y, Nomura S, Kawasaki S, Tsutsumimoto T, Shimizu T, Takaoka K (2001). Colocalization of noggin and bone morphogenetic protein-4 during fracture healing. J Bone Miner Res.

[21] Niikura T, Hak DJ, Reddi AH (2006). Global gene profiling reveals a downregulation of BMP gene expression in experimental atrophic nonunions compared to standard healing fractures. J Orthop Res.

[22] Marsell R, Einhorn TA (2009). The role of endogenous bone morphogenetic proteins in normal skeletal repair. Injury.

[23] Govender S, Csimma C, Genant HK, Valentin-Opran A, Amit Y, Arbel R (2002). Recombinant human bone morphogenetic protein-2 for treatment of open tibial fractures: a prospective, controlled, randomized study of four hundred and fifty patients. J Bone Joint Surg Am.

[24] Friedlaender GE, Perry CR, Cole JD, Cook SD, Cierny G, Muschler GF (2001). Osteogenic protein-1 (bone morphogenetic protein-7) in the treatment of tibial nonunions. J Bone Joint Surg Am.

[25] Miska M, Findeisen S, Tanner M, Biglari B, Studier-Fischer S, Grützner PA (2016). Treatment of nonunions in fractures of the humeral shaft according to the Diamond Concept. Bone Joint J.

[26] Moghaddam A, Zietzschmann S, Bruckner T, Schmidmaier G (2015). Treatment of atrophic tibia non-unions according to ‘diamond concept’: results of one- and two-step treatment. Injury.

[27] Giannoudis PV, Gudipati S, Harwood P, Kanakaris NK (2015). Long bone non-unions treated with the diamond concept: a case series of 64 patients. Injury.

[28] Andrzejowski P, Giannoudis PV (2019). The ‘diamond concept’ for long bone non-union management. J Orthop Traumatol.

[29] Gruber HE, Ode G, Hoelscher G, Ingram J, Bethea S, Bosse MJ (2016). Osteogenic, stem cell and molecular characterisation of the human induced membrane from extremity bone defects. Bone Joint Res.

[30] Henrich D, Seebach C, Nau C, Basan S, Relja B, Wilhelm K (2016). Establishment and characterization of the Masquelet induced membrane technique in a rat femur critical-sized defect model. J Tissue Eng Regen Med.

[31] Tetsworth K, Woloszyk A, Glatt V (2019). 3D printed titanium cages combined with the Masquelet technique for the reconstruction of segmental femoral defects: preliminary clinical results and molecular analysis of the biological activity of human-induced membranes. OTA Int.

[32] Haines NM, Lack WD, Seymour RB, Bosse MJ (2016). Defining the lower limit of a ‘critical bone defect’ in open diaphyseal tibial fractures. J Orthop Trauma.

[33] Careri S, Vitiello R, Oliva MS, Ziranu A, Maccauro G, Perisano C (2019). Masquelet technique and osteomyelitis: innovations and literature review. Eur Rev Med Pharmacol Sci.

[34] Niikura T, Jimbo N, Komatsu M, Oe K, Fukui T, Matsumoto T (2021). Histological analysis of induced membranes in patients whose bone defects were treated with the Masquelet technique to identify factors affecting the vascularity of induced membranes. J Orthop Surg Res.

[35] Vitiello R, Bocchi MB, Gessi M, Greco T, Cianni L, de Maio F (2020). Induced membrane by silver-coated knee megaprosthesis: keep or toss?. J Biol Regul Homeost Agents.

[36] Bosemark P, Perdikouri C, Pelkonen M, Isaksson H, Tägil M (2015). The masquelet induced membrane technique with BMP and a synthetic scaffold can heal a rat femoral critical size defect. J Orthop Res.

[37] Haubruck P, Tanner MC, Vlachopoulos W, Hagelskamp S, Miska M, Ober J (2018). Comparison of the clinical effectiveness of bone morphogenic protein (BMP) −2 and −7 in the adjunct treatment of lower limb nonunions. Orthop Traumatol Surg Res.

[38] Major Extremity Trauma Research Consortium (METRC). A randomized controlled trial comparing rhBMP-2/Absorbable collagen sponge versus autograft for the treatment of tibia fractures with critical size defects. J Orthop Trauma. 2019;33(8):384–91 10.1097/BOT.0000000000001492 31022069

[39] Bilic R, Simic P, Jelic M, Stern-Padovan R, Dodig D, van Meerdervoort HP (2006). Osteogenic protein-1 (BMP-7) accelerates healing of scaphoid non-union with proximal pole sclerosis. Int Orthop.

[40] Giannoudis PV, Kanakaris NK, Dimitriou R, Gill I, Kolimarala V, Montgomery RJ (2009). The synergistic effect of autograft and BMP-7 in the treatment of atrophic nonunions. Clin Orthop Relat Res.

[41] Crawford CH, Seligson D (2009). Atrophic nonunion of humeral diaphysis treated with locking plate and recombinant bone morphogenetic protein: nine cases. Am J Orthop (Belle Mead NJ).

[42] von Rüden C, Morgenstern M, Friederichs J, Augat P, Hackl S, Woltmann A (2016). Comparative study suggests that human bone morphogenetic proteins have no influence on the outcome of operative treatment of aseptic clavicle non-unions. Int Orthop.

[43] Song K, Krause C, Shi S, Patterson M, Suto R, Grgurevic L (2010). Identification of a key residue mediating bone morphogenetic protein (BMP)-6 resistance to noggin inhibition allows for engineered BMPs with superior agonist activity. J Biol Chem.

[44] Vukicevic S, Oppermann H, Verbanac D, Jankolija M, Popek I, Curak J (2014). The clinical use of bone morphogenetic proteins revisited: a novel biocompatible carrier device OSTEOGROW for bone healing. Int Orthop.

[45] Vukičević S, Grgurević L, Pećina M (2017). Clinical need for bone morphogenetic proteins. Int Orthop.

[46] Bharadwaz A, Jayasuriya AC (2021). Osteogenic differentiation cues of the bone morphogenetic protein-9 (BMP-9) and its recent advances in bone tissue regeneration. Mater Sci Eng C Mater Biol Appl.

[47] Wang X, Huang J, Huang F, Zong JC, Tang X, Liu Y (2017). Bone morphogenetic protein 9 stimulates callus formation in osteoporotic rats during fracture healing. Mol Med Rep.

[48] Mostafa S, Pakvasa M, Coalson E, Zhu A, Alverdy A, Castillo H (2019). The wonders of BMP9: from mesenchymal stem cell differentiation, angiogenesis, neurogenesis, tumorigenesis, and metabolism to regenerative medicine. Genes Dis.

